# Need for standardization in norepinephrine dosage in the management of septic shock

**DOI:** 10.17843/rpmesp.2025.424.15450

**Published:** 2025-12-11

**Authors:** Jorge Cerna, Liz Aliaga, José Amado, Daisy Torres, Patricia Martínez, Rosa Lisson, Consuelo Cornejo, Arturo Ota

**Affiliations:** 1 Hospital Nacional Edgardo Rebagliati Martins, Lima, Peru. Hospital Nacional Edgardo Rebagliati Martins Lima Peru; 2 Facultad de Medicina, Universidad Ricardo Palma, Lima, Peru. Universidad Ricardo Palma Facultad de Medicina Universidad Ricardo Palma Lima Peru; 3 Facultad de Medicina, Universidad Nacional Mayor de San Marcos, Lima, Peru. Universidad Nacional Mayor de San Marcos Facultad de Medicina Universidad Nacional Mayor de San Marcos Lima Peru

Mr. Editor. Septic circulatory shock is one of the most frequent causes of admission to Emergency Departments and Intensive Care Units worldwide ^1^. Its mortality ranges between 45% and 60% according to patient age, despite the advances achieved in the last 20 years in early treatment with fluids, antibiotics, and vasopressors ^2^. The use of norepinephrine as an inotropic/vasopressor drug is indicated as the first option to improve hemodynamic status, in conjunction with fluid therapy.

The different clinical guidelines for the management of septic shock establish ranges for initial and maximum doses of norepinephrine, as well as the possibility of adding another vasopressor when the response is insufficient. However, these clinical guidelines do not specify to which pharmaceutical presentation of norepinephrine the recommended doses correspond ^3^^-^^5^. In 2022, Leone and collaborators, in their regional and global review of 32 world pharmacopoeias, described the different presentations of norepinephrine available for clinical use, mentioning tartrate, bitartrate, and hydrochloride salts for parenteral use. The equivalence with respect to base norepinephrine varies according to the salt used: 1.9 mg of tartrate, 2 mg of bitartrate, and 0.9 mg of hydrochloride are equivalent to 1 mg of base norepinephrine ^3^.

Consequently, the dose of base norepinephrine is equivalent to approximately half the dose of its bitartrate salt, which has a significant impact on clinical practice and affects the doses used in clinical practice. For example, a 45-year-old male patient, weighing 70 kg, diagnosed with septic shock, starts an intravenous infusion of norepinephrine. If two ampoules of norepinephrine bitartrate 4 mg in 100 ml of 0.9% saline solution are administered at an infusion rate of 10 ml/h, the administered dose would be 0. μg/kg/min. But this is equivalent to 0.09 μg/kg/min of base norepinephrine, a value lower than the range recommended in clinical practice guidelines (0.5 to 1 ug/kg/min), which could lead to insufficient treatment.

In Peru, according to the General Directorate of Medicines, Supplies, and Drugs (DIGEMID), as of 2025, only one pharmacological presentation of norepinephrine bitartrate is officially registered (Table 1). However, upon reviewing the information corresponding to this drug, the declared composition is not entirely clear. This lack of precision, added to the absence of an explicit dose in clinical practice guidelines, could lead to prescription errors.

**Table 1:** Data on norepinephrine drug presentations approved in Peru, 2025.

Sanitary Registration	Name	Pharmaceutical Form	Declared Composition
EE08899	Norepinephrine 4 mg/4 mL	Solution for injection	Norepinephrine bitartrate 7.96 mg
EE08999	Norepinephrine 4 mg/4 mL	Concentrate for solution for infusion	Norepinephrine bitartrate 4.00000 mg.
EE10006	Norepinephrine 4 mg/4 mL	Concentrate for solution for infusion.	Norepinephrine bitartrate monohydrate 1.00000 mg.
EE10124	PINEPHED 4mg/ml	Concentrate for solution for infusion.	Norepinephrine bitartrate 8.0000 mg.
EN00949	Norepinephrine 4 mg/4 mL	Solution for injection	Norepinephrine bitartrate 7.574000 mg.
EN02831	Norepinephrine 4 mg/4 mL	Solution for injection	Norepinephrine bitartrate 7.514000 mg.
EN06417	NIACOR 4mg/ml	Concentrate for solution for infusion	Norepinephrine bitartrate monohydrate 7.974000 mg.
EN04970	Norepinephrine 4 mg/4 mL	Concentrate for solution for infusion	Norepinephrine bitartrate 7.974000 mg.

Source: https://www.digemid.minsa.gob.pe/rsProductosFarmaceuticos/

Therefore, it is recommended that both clinical practice guidelines and the technical data sheets of commercialized products explicitly use base norepinephrine as a reference ^6^. Given that this information is of great relevance for clinical practice and research, we call on researchers and clinical practice guideline developers to consider this recommendation in order to optimize the therapeutic management of patients.
