# Supplementary material for: Need for standardization in norepinephrine dosage in the management of septic shock
Source: Rev Peru Med Exp Salud Publica. 2025 Dec 11;42(4):458–9. doi: 10.17843/rpmesp.2025.424.15450 (PMC12879974; doi:10.17843/rpmesp.2025.424.15450)
Supplement: Translation

# Translation: Necesidad de estandarización en la dosificación de norepinefrina en el manejo del choque séptico

**Authors:** Jorge Cerna, Liz Aliaga, José Amado, Daisy Torres, Patricia Martínez, Rosa Lisson, Consuelo Cornejo, Arturo Ota

**Affiliations:** 1 Hospital Nacional Edgardo Rebagliati Martins, Lima, Perú.; 2 School of Medicine, Universidad Ricardo Palma, Lima, Perú.; 3 School of Medicine, Universidad Nacional Mayor de San Marcos, Lima, Perú.

Sr. Editor. El choque circulatorio séptico es una de las causas más frecuentes de ingreso a las Emergencias y Unidades de Cuidados Intensivos en el Mundo ^1^. Su mortalidad oscila entre 45% y 60% de acuerdo a la edad del paciente, a pesar de los avances logrados en los últimos 20 años en el tratamiento temprano con fluidos, antibióticos y vasopresores ^2^. El uso de norepinefrina como fármaco inotrópico/vasopresor está indicado como primera opción para mejorar el estado hemodinámico, en conjunto con la fluidoterapia.

Las diferentes guías clínicas para el manejo de choque séptico establecen rangos de dosis iniciales y máximas de norepinefrina, así como la posibilidad de añadir otro vasopresor cuando la respuesta es insuficiente. Sin embargo, dichas guías clínicas no especifican a que presentación farmacéutica de norepinefrina corresponden las dosis recomendadas ^3^^-^^5^. En el año 2022, Leone y colaboradores en su revisión a nivel regional y global de 32 farmacopeas del mundo, describieron las diferentes presentaciones de la norepinefrina disponibles para su uso clínico mencionando sales de tartrato, bitartrato e hidrocloruro, para uso parenteral. La equivalencia respecto a la norepinefrina base varía de acuerdo a la sal empleada: 1,9 mg de tartrato, 2 mg de bitartrato y 0,9 mg de hidrocloruro equivalen a 1 mg de norepinefrina base ^3^.

Por consiguiente, la dosis de norepinefrina base equivale aproximadamente la mitad de la dosis de su sal bitartrato, lo cual tiene un impacto significativo en la práctica clínica y esto tiene un efecto en las dosis utilizadas en la práctica clínica. Por ejemplo, un paciente varón de 45 años, con un peso de 70 kg, con diagnóstico de choque séptico, inicia infusión endovenosa de norepinefrina. Si se administran dos ampollas de norepinefrina bitartrato 4 mg en 100 ml de solución salina al 0,9%, a una velocidad de infusión de 10 ml/h, la dosis administrada sería de 0,18 μg/kg/min. Pero esto equivale a 0,09 μg/kg/min de la norepinefrina base, valor inferior al rango recomendado en las guías de práctica clínica (0,5 a 1 ug/kg/min), lo que podría conducir a un tratamiento insuficiente.

En el Perú según la Dirección General de Medicamentos, Insumos y Drogas (DIGEMID), al año 2025, solo se dispone de una presentación farmacológica del bitartrato de norepinefrina registrada oficialmente (Tabla 1). Sin embargo, al revisar la información correspondiente a dicho fármaco, la composición declarada no resulta del todo clara. Esta falta de precisión, sumada a la ausencia de una dosis explícita en las guías de práctica clínica, podría conducir a errores en la prescripción.

**Tabla 1:** Datos de las presentaciones farmacológicas de norepinefrina aprobadas en el Perú, 2025.

Registro sanitario	Nombre	Forma farmacéutica	Composición declarada RS
EE08899	Norepinefrina 4mg/4ml	Solución inyectable	Bitartrato de norepinefrina 7,96 mg
EE08999	Norepinefrina 4mg/4ml	Concentrado para solución para perfusión	Bitartrato de norepinefrina 4.00000 mg
EE10006	Norepinefrina 4mg/4ml	Concentrado para solución para perfusión	Bitartrato de norepinefrina monohidrato 1.00000 mg
EE10124	PINEPHED 4mg/ml	Concentrado para solución para perfusión	Bitartrato de norepinefrina 8.0000 mg
EN00949	Norepinefrina 4mg/ml	Solución inyectable	Bitartrato de norepinefrina 7.574000 mg
EN02831	Norepinefrina 4mg/ml	Solución inyectable	Bitartrato de norepinefrina 7.514000 mg
EN06417	NIACOR 4mg/ml	Concentrado para solución para perfusión	Bitartrato de norepinefrina monohidrato 7.974000 mg
EN04970	Norepinefrina 4mg/ml	Concentrado para solución para perfusión	Bitartrato de norepinefrina 7.974000 mg

Fuente: https://www.digemid.minsa.gob.pe/rsProductosFarmaceuticos/.

Por ello, se recomienda que tanto las guías de práctica clínica como las fichas técnicas de los productos comercializados utilicen como referencia explícita la norepinefrina base ^6^. Dado que esta información es de gran relevancia para la práctica clínica y la investigación, hacemos un llamado a los investigadores y a los elaboradores de guías de prácticas clínica a considerar esta recomendación con el fin de optimizar el manejo terapéutico de los pacientes.
